# The Efficacy of Adjuvant Corticosteroids in Surgical Management of Chronic Subdural Hematoma: A Systematic Review and Meta-Analysis

**DOI:** 10.3389/fneur.2021.744266

**Published:** 2022-01-13

**Authors:** Guoqiang Tang, Jiabei Chen, Bin Li, Song Fang

**Affiliations:** Department of Neurosurgery, Chenzhou No.1 People's Hospital, Chenzhou, China

**Keywords:** intracranial hemorrhage, subdural hematoma, steroids, surgery, recurrence

## Abstract

**Objective:** This systematic review aimed to assess the efficacy of adjuvant corticosteroids in managing patients with chronic subdural hematoma (CSDH) undergoing surgical intervention.

**Methods:** We searched for eligible studies electronically on the databases of PubMed, Embase, and Google Scholar. The last date of the search was 15th Jun 2021. Outcomes were pooled to calculate risk ratios (RR) with 95% confidence intervals (CI).

**Results:** Eleven studies were included. Four of them were randomized controlled trials (RCTs). Six studies reported data on good neurological outcomes but with variable definitions. Combining all studies, we noted no statistically significant difference in good neurological outcome with the use of adjuvant corticosteroids (RR: 0.91 95% CI: 0.74, 1.12 *I*^2^ = 92% *p* = 0.39). Similar results were obtained on subgroup analysis based on definition and study type. However, the use of adjuvant corticosteroids was associated with a significantly reduced risk of recurrence (RR: 0.51 95% CI: 0.40, 0.64 *I*^2^ = 0% *p* < 0.0001). The meta-analysis also demonstrated no statistically significant difference in mortality rates with the use of adjuvant corticosteroids (RR: 1.01 95% CI: 0.47, 2.21 *I*^2^ = 76% *p* = 0.97). The results did not differ between RCTs and non-RCTs. Limited studies reported data on complications, and pooled analysis indicated no significant increase in infectious, gastrointestinal, and neurological complications with the use of adjuvant corticosteroids.

**Conclusion:** The use of corticosteroids with surgery for CSDH might be associated with a reduction in recurrence rate. However, corticosteroids do not improve functional outcomes or mortality rates. Future studies should assess the impact of different corticosteroid regimens on patient outcomes, and should use standardized reporting of neurological outcomes with uniform follow-up duration.

## Introduction

Chronic subdural hematoma (CSDH) is one of the most frequent indications for neurosurgical intervention. The disease is characterized by an abnormal collection of blood in the subdural space, and is slow in onset and progression (1). Due to the increase in the elderly population along with higher trends of antiplatelet and anticoagulant prescriptions, the incidence of CSDH is significantly higher in older adults (2). Estimates suggest an incidence of 15 per 100,000 person-years in the general population, increasing to 127.1 per 100,000 person-years in elderly patients (3). While CSDH has a favorable outcome with adequate management, it can also lead to significant morbidity and mortality. Rauhala et al. (4) in a recent study have suggested that CSDH leads to excess mortality rates of 18% at 5 years and 48% at 20 years.

Surgical interventions are usually recommended in patients with CSDH that demonstrate neurological symptoms (5). Of them, burr-hole craniostomy is considered the most popular surgical procedure that results in good neurological outcomes (6). However, even after appropriate management, recurrence continues to be a major problem with a frequency ranging from 3 to 30% (7, 8). It is to be noted that the mass effect of the hematoma is reduced by surgical evacuation, but it does not treat the underlying pathophysiological mechanisms. In this context, several adjuvant therapies like the use of atorvastatin, angiotensin-converting enzyme inhibitors, corticosteroids, and middle meningeal artery embolization have been used to manage patients with CSDH (9–12). While the use of corticosteroids is popular, their efficacy is still unclear. In a systematic review and meta-analysis, Holl et al. (9) attempted to analyze evidence on the efficacy of corticosteroids in the management of CSDH. In their meta-analysis, they conducted a three-way comparison of the efficacy of corticosteroids, surgery, and corticosteroids plus surgery for managing CSDH. However, the analysis only included six studies that compared the use of corticosteroids with surgery vs. surgery alone. Moreover, of these six studies, only one was a randomized controlled trial (RCT). With the recent publication of several new studies (13–15), there is a need for more updated and comprehensive evidence to guide clinical practice. Thus, the current review aimed to perform a systematic literature search and pool evidence on the efficacy of adjuvant corticosteroids in improving outcomes of CSDH patients undergoing surgical intervention.

## Materials and Methods

### Research Question

We aimed to answer the following research question: does the use of corticosteroids as an adjuvant to surgery improves outcomes in patients with CSDH? This review was conducted based on the guidelines of the PRISMA statement (Preferred Reporting Items for Systematic Reviews and Meta-analyses) (16). The review protocol was registered on PROSPERO (No CRD42021258308). The protocol was registered to compare outcomes of CSDH patients with two additional subgroups: corticosteroids alone vs. surgery alone and corticosteroids alone vs. corticosteroids and surgery. However, after the final literature search, we could not find any new studies to add to already published data. Hence, the current review focused only on comparing outcomes with surgery alone vs. corticosteroids and surgery for CSDH.

### Literature Search

Systematic search for eligible studies was conducted in PubMed, Embase, and Google Scholar electronic databases independently by two reviewers. Search limits were from the inception of the databases to 15th Jun 2021. The search was restricted to only English language studies. The main terms used for the literature search in various combinations were: “chronic subdural hematoma,” “intracranial hemorrhage,” “corticosteroids,” “dexamethasone,” “prednisone,” “surgery,” “craniostomy,” and “burr-hole.” The details of the search strategy are summarized in Supplementary Table 1. After excluding duplicates, we reviewed the output of each database by assessing the titles and abstracts of every study. We identified articles relevant to the review and extracted their full texts. The two reviewers independently evaluated these studies for the final inclusion in the review. We resolved any disagreements by discussion. In the end, we reviewed the reference list of the included studies for any missed references.

### Eligibility Criteria

The eligibility criteria were based on the PICOS (Population, Intervention, Comparison, Outcome, Study type) framework. We included (1) All prospective or retrospective cohort studies or RCTs that were carried out on patients undergoing surgical intervention for newly diagnosed supratentorial CSDH (*Population*). (2) Studies that had an *Intervention* group of patients receiving corticosteroids and a *Comparative* group of patients receiving placebo or no corticosteroid. (3) Studies that assessed at least one of the following *Outcomes*: good neurological outcomes, recurrence, mortality, or complications. We did not pre-define the criteria for good neurological outcome and used the definition from the included studies. During the literature search we identified some studies comparing corticosteroids with placebo but including a mix of surgical and conservatively managed CSDH patients. We decided to also include those studies if >90% of the cohort underwent surgical intervention.

Exclusion criteria for the review were as follows: (1) Studies on patients undergoing only conservative management of CSDH (2) Studies not including at least 10 patients in each arm (3) Studies not comparing outcomes with control group (4) Case series, case reports, and review articles. (5) Studies reporting duplicate data. In case of two or more studies from the same healthcare setup, we included the article with the largest sample size.

### Data Extraction and Quality Assessment

Data from each study was sourced by two authors independently. We extracted details of the first author, publication year, study type, study location, sample size, mean age, gender, preoperative Glasgow coma scale (GCS), number of patients with preoperative GCS <12 or Markwalder Grading Scale (MGS) grade 3 and above, midline shift, drain placement, dosage, and protocol of corticosteroids, the definition of good neurological outcome, follow-up duration, and study outcomes. The outcomes of interest were rates of good neurological outcomes, recurrence of CSDH, mortality, and complications. Studies reporting reintervention rates instead of recurrence were also included in the meta-analysis for recurrence.

The quality of included studies was assessed independently by two study investigators. The risk of a bias assessment tool for non-randomized studies (RoBANS) was used for non-RCTs (17). Studies were rated as low risk, high risk, or unclear risk of bias for selection of participants, confounding variables, intervention measurements, blinding of outcome assessment, incomplete outcome data, and selective outcome reporting. The recent Cochrane Collaboration's risk of bias assessment tool-2 was used to assess the quality of the included RCTs (18). The following five domains were used for quality assessment: randomization process, deviation from intended intervention, missing outcome data, measurement of outcomes, and selection of reported results. Based on the risk of bias in individual domains, the overall bias was marked as “high risk,” “some concerns,” or “low risk.” Any disagreements related to data extraction or quality assessment were resolved by discussion.

### Statistical Analysis

The meta-analysis was conducted using “Review Manager” (RevMan, version 5.3; Nordic Cochrane Centre [Cochrane Collaboration], Copenhagen, Denmark; 2014). We used a random-effects model for all outcomes. Data was pooled using risk ratios (OR) with 95% confidence intervals (CI). A sensitivity analysis was also performed wherein individual studies were sequentially excluded from the meta-analysis in the software itself to check any undue influence of the study on the total effect size. A sub-group analysis was performed based on the definition of good neurological outcome and study type. Heterogeneity was assessed using the *I*^2^ statistic. *I*^2^ values of 25–50% represented low, 50–75% medium, and >75% substantial heterogeneity. We used funnel plots to assess publication bias. *P* ≤ 0.05 was considered statistically significant.

## Results

**Figure 1:**
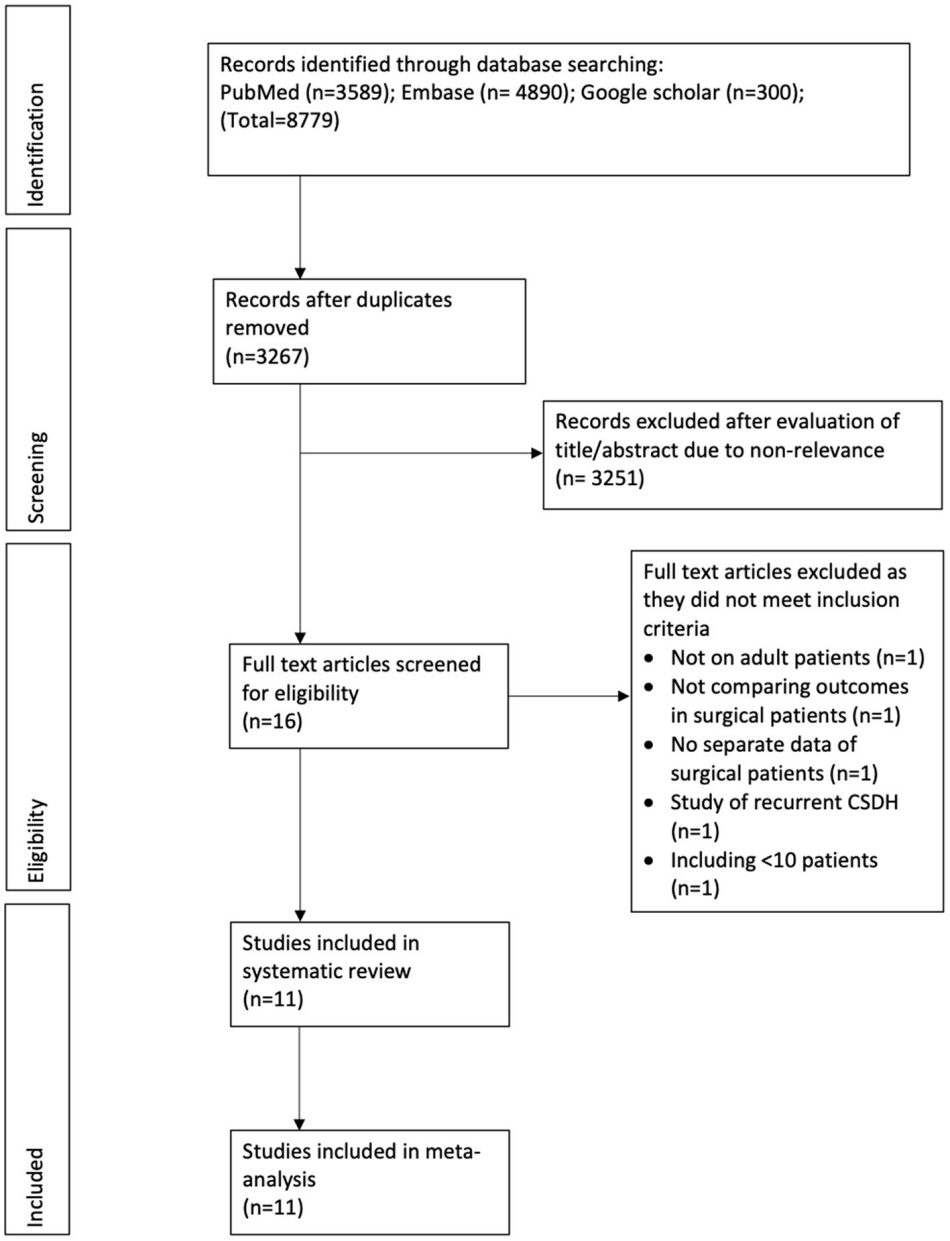
Study flow chart.

The study flow chart is presented in Figure 1. A total of 11 studies met the inclusion criteria (13–15, 19–26). In all the included studies 100% of patients underwent surgical intervention in both intervention and control arms, except for one. The trial of Hutchinson et al. (19) was included as 94% of their patients underwent surgery. Details of the included studies are summarized in Table 1. Four of the included studies were RCTs (13, 15, 19, 22), one was a prospective cohort study (25) while the remaining were retrospective in nature. The sample size of the adjuvant corticosteroid group ranged from 23 to 437 patients, while that of the surgical group ranged from 13 to 375 patients. The mean age of patients was >70 years in most studies. Limited data were available on mean preoperative GCS or MGS grade. All studies used dexamethasone except two which used prednisone (13) and methylprednisolone (MP) (23). The starting dose of dexamethasone ranged from 8 to 16 mg/day. Prednisone was used with a starting dose of 1 mg/kg/day while MP was used at 0.5 mg/kg/day. Most studies reported outcomes after a follow-up of 6 months. The follow-up was just 30 days in one retrospective study (14). Two studies (13, 23) reported outcomes after 1 year of follow-up.

**Table 1:** Details of included studies.

**Study**	**Location**	**Type**	**Groups**	**Sample size**	**Mean age**	**Male gender (%)**	**Mean preoperative GCS**	**No with GCS <12**	**No with MGC >3 (%)**	**Midline shift (mm)**	**No drain (%)**	**Starting dose of steroid**	**Tapering course (days)**	**Follow-up**
Sun et al. (25)	Hong Kong	P	S CS	13 69	NR	NR	NR	2 8	NR	NR	100 100	DXM 16 mg/day	2–21	6 months
Dran et al. (23)	France	R	S CS	56 142	77.4 74	NR	NR	NR	16 10	NR	0 0	MP 0.5 mg/kg/ day	>1 month	17.5 months
Delgado et al. (26)	Spain	R	S CS	19 25	NR	NR	NR	NR	10.5 4	NR	0 0	DXM 12 mg/day	36 days	25 weeks
Chan et al. (22)	China	RCT	S CS	126 122	70.8 71.8	69.8 73	NR	NR	2.4 2.5	NR	NR	DXM 16 mg/day	10 days	6 months
Qian et al. (21)	China	R	S CS	167 75	NR	NR	NR	NR	NR	NR	0 0	DXM 12 mg/day	40 days	6 months
Fountas et al. (20)	Greece	R	S CS	136 25	76.9 75.5	67.6 80	14 ± 1 14 ± 1	NR	NR	8 ± 4.3 6.4 ± 4.6	0 0	DXM 24 mg/day	14 days	>3 months
Mebberson et al. (15)	Australia	RCT	S CS	24 23	75.1 73.3	79 65	NR	NR	NR	7.2 ± 4.6 6.4 ± 4.1	0 0	DXM 16 mg/day	14 days	6 months
Cofano et al. (24)	Italy	R	S CS	282 437	NR	NR	NR	NR	NR	NR	NR	DXM 8 mg/day	NR	NR
Hutchinson et al. (19)	UK	RCT	S CS	375 373	74.3 74.5	76.7 71.5	NR	21 21	40.1 40*	NR	15 17.4	DXM 16 mg/day	14 days	6 months
Lodewijkx et al. (14)	Netherlands	R	S CS	247 278	73 75	74 76	15 [14–15] 14 [14–15]	NR	NR	8 ± 5 9 ± 5	6 8	DXM 15 mg/day	Up to 29 days	30 days
Ng et al. (13)	France	RCT	S CS	77 78	72.7 75.6	75.3 71.8	NR	NR	NR	NR	1.3 1.3	Prednisone 1 mg/kg/day	21 days	12 months

*Median [interquartile range]*.

* *Data of MGC <4*.

*R, retrospective; P, prospective; RCT, randomized controlled trial; S, surgery; CS, corticosteroid and surgery; NR, not reported; GCS, Glasgow coma scale; DXM, dexamethasone, MP, methylprednisolone; MGC, Markwalder Grading Scale*.

### Good Neurological Outcome

**Figure 2:**
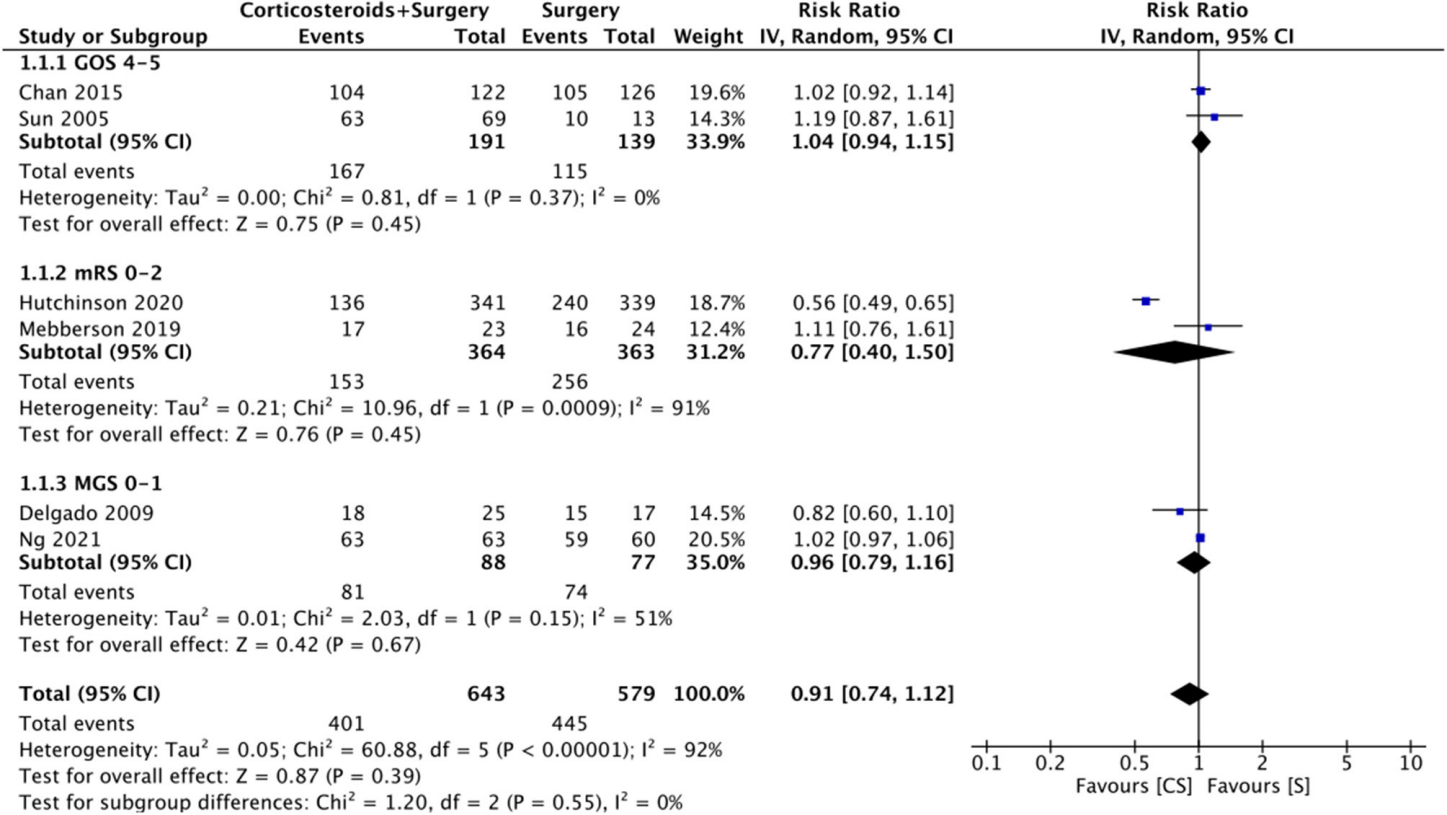
Meta-analysis of good neurological outcome between corticosteroid and surgery (CS) and surgery (S) groups with subgroup analysis based on the definition of good neurological outcome.

**Figure 3:**
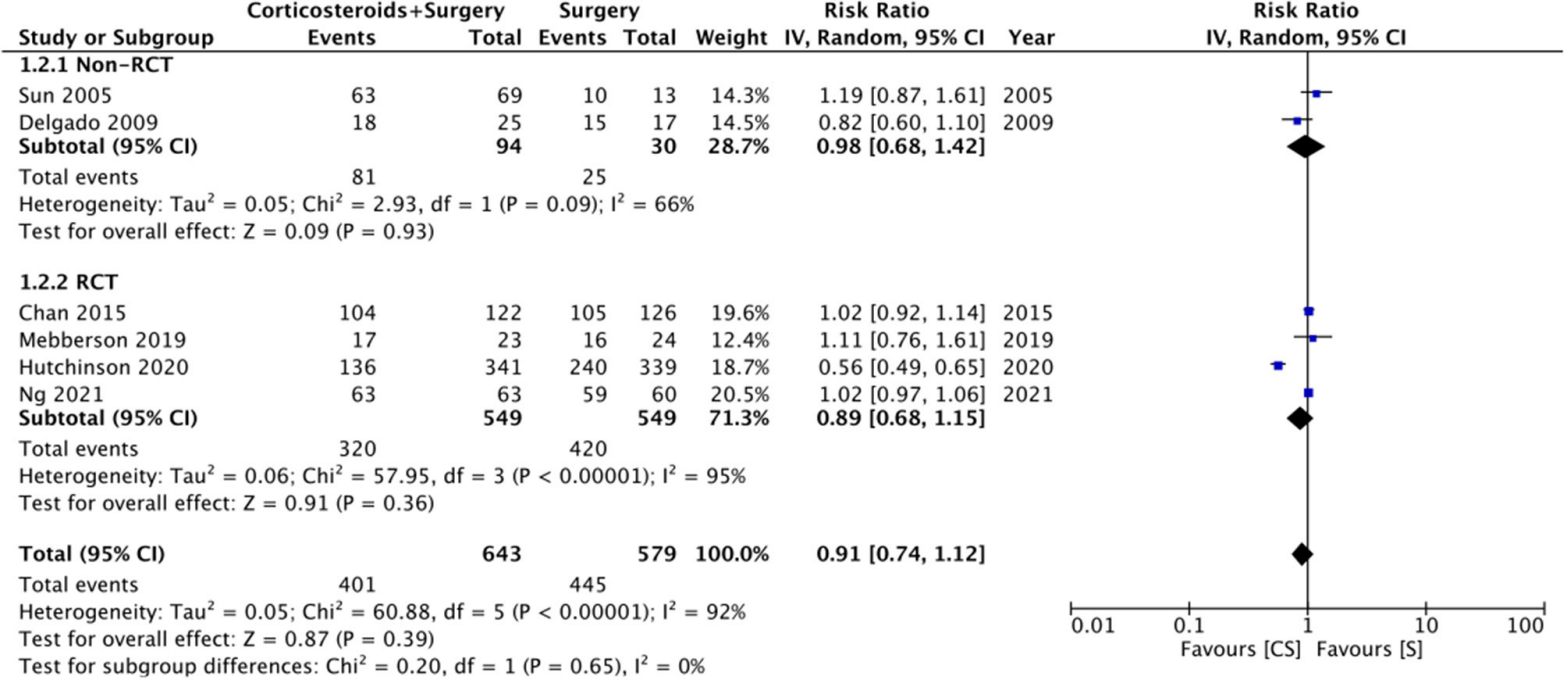
Meta-analysis of good neurological outcome between corticosteroid and surgery (CS) and surgery (S) groups with subgroup analysis based on study type.

Six studies reported data on good neurological outcomes but with variable definitions. It was defined as either Glasgow outcome scale (GOS) of 4–5, modified Ranking Scale (mRS) of 0–2, or MGS grade 0–1 at the end of the follow-up period. Combining all studies, we noted no statistically significant difference in good neurological outcomes associated with the use of adjuvant corticosteroids (RR: 0.91 95% CI: 0.74, 1.12 *I*^2^ = 92% *p* = 0.39) (Figure 2). The difference was non-significant even on subgroup analysis based on the definition; GOS (RR: 1.04 95% CI: 0.94, 1.15 *I*^2^ = 0% *p* = 0.45), mRS (RR: 0.77 95% CI: 0.40, 1.50 *I*^2^ = 91% *p* = 0.45), and MGS (RR: 0.96 95% CI: 0.79, 1.16 *I*^2^ = 51% *p* = 0.67) (Figure 2). We also noted no beneficial effect of adjuvant corticosteroids when data of non-RCTs (RR: 0.98 95% CI: 0.68, 1.42 *I*^2^ = 66% *p* = 0.93) and RCTs (RR: 0.89 95% CI: 0.68, 1.15 *I*^2^ = 95% *p* = 0.36) were pooled separately (Figure 3). The results did not change with the exclusion of any study during the sensitivity analysis. There was no evidence of publication bias (Supplementary Figure 1).

### Recurrence

**Figure 4:**
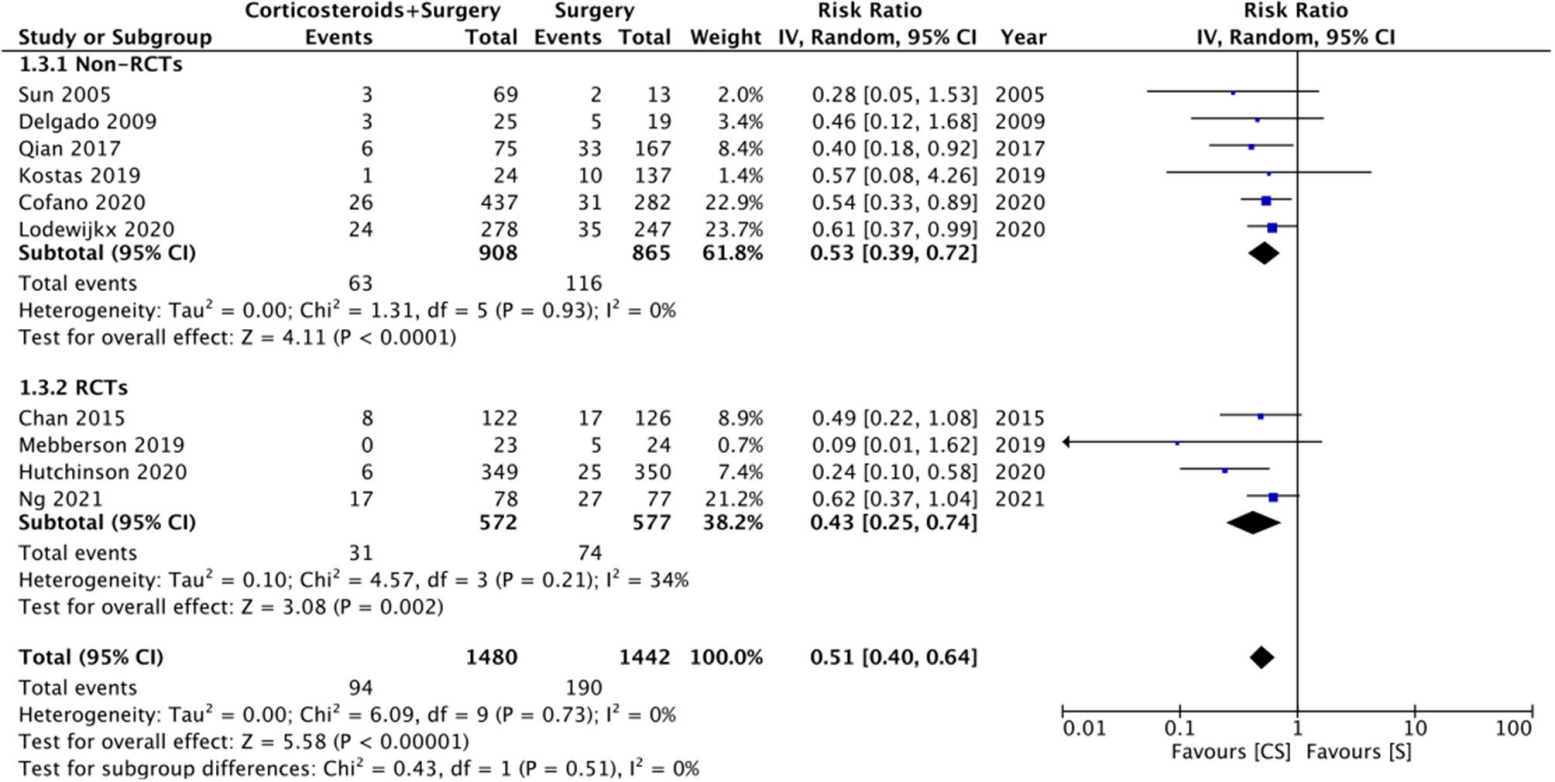
Meta-analysis of recurrence between corticosteroid and surgery (CS) and surgery (S) groups.

Ten studies reported data on CSDH recurrence. On pooled analysis, the use of adjuvant corticosteroids was associated with a significantly reduced risk of recurrence (RR: 0.51 95% CI: 0.40, 0.64 *I*^2^ = 0% *p* < 0.0001) (Figure 4). There was no change in the significance of results with the exclusion of any study during the sensitivity analysis. On subgroup analysis for recurrence rates based on study type, we noted no statistically significant difference between RCTs (RR: 0.43 95% CI: 0.25, 0.74 *I*^2^ = 34% *p* = 0.02) and non-RCTs (RR: 0.53 95% CI: 0.39, 0.72 *I*^2^ = 0% *p* < 0.0001). There was no evidence of publication bias (Supplementary Figure 2).

### Mortality

**Figure 5:**
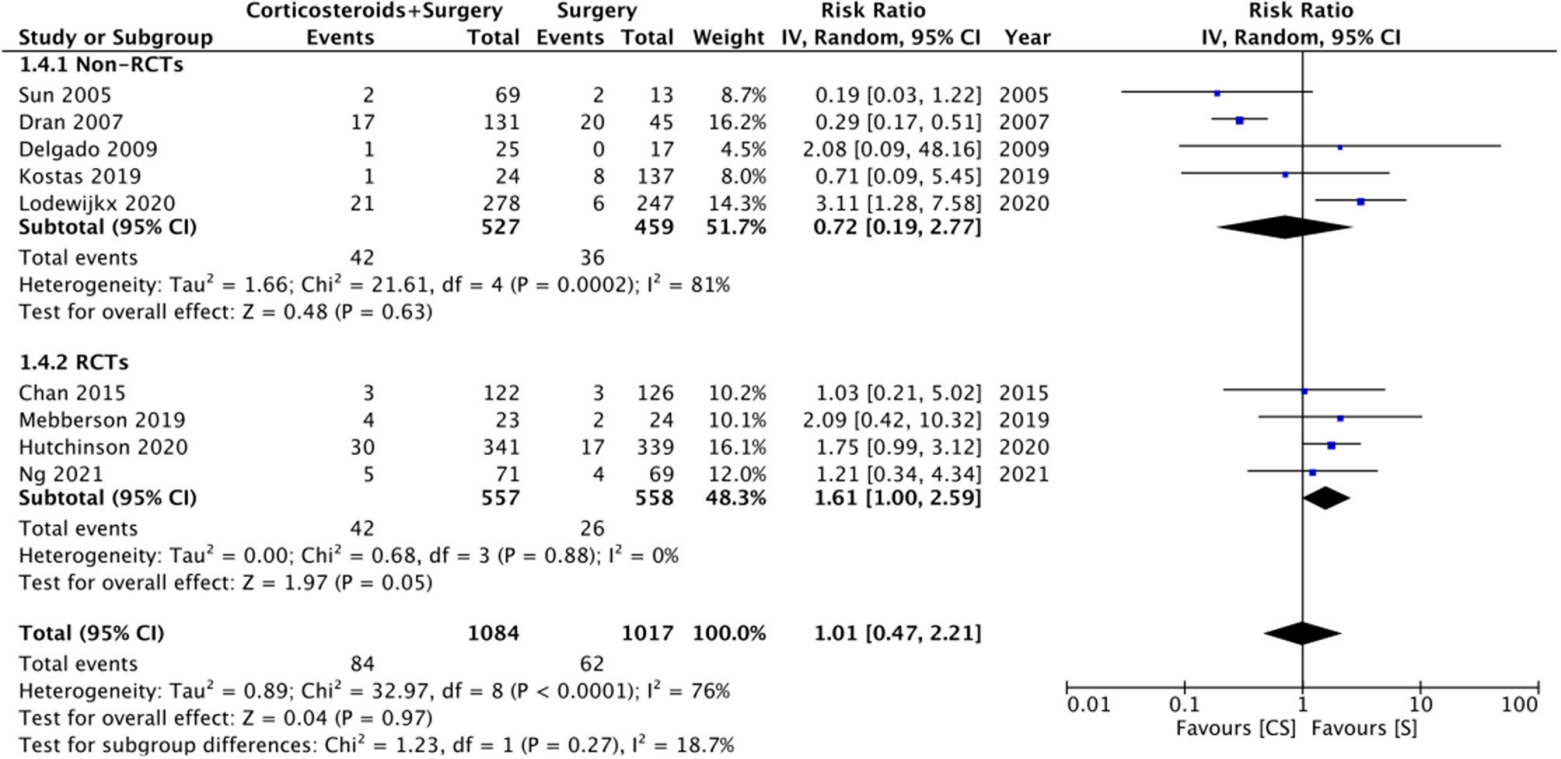
Meta-analysis of mortality between corticosteroid and surgery (CS) and surgery (S) groups with subgroup analysis based on study type.

Nine studies reported data on mortality rates but at different follow-up times. Meta-analysis demonstrated no statistically significant difference in mortality rates with the use of adjuvant corticosteroids (RR: 1.01 95% CI: 0.47, 2.21 *I*^2^ = 76% *p* = 0.97) (Figure 5). Results were similar on subgroup analysis of RCTs (RR: 1.61 95% CI: 1.00, 2.59 *I*^2^ = 0% *p* = 0.05) and non-RCTs (RR: 0.72 95% CI: 0.19, 2.77 *I*^2^ = 81% *p* = 0.63) (Figure 5). There was no change in the significance of the results on sensitivity analysis. We noted no evidence of publication bias on visual inspection of the funnel plot (Supplementary Figure 3).

### Complications

**Figure 6:**
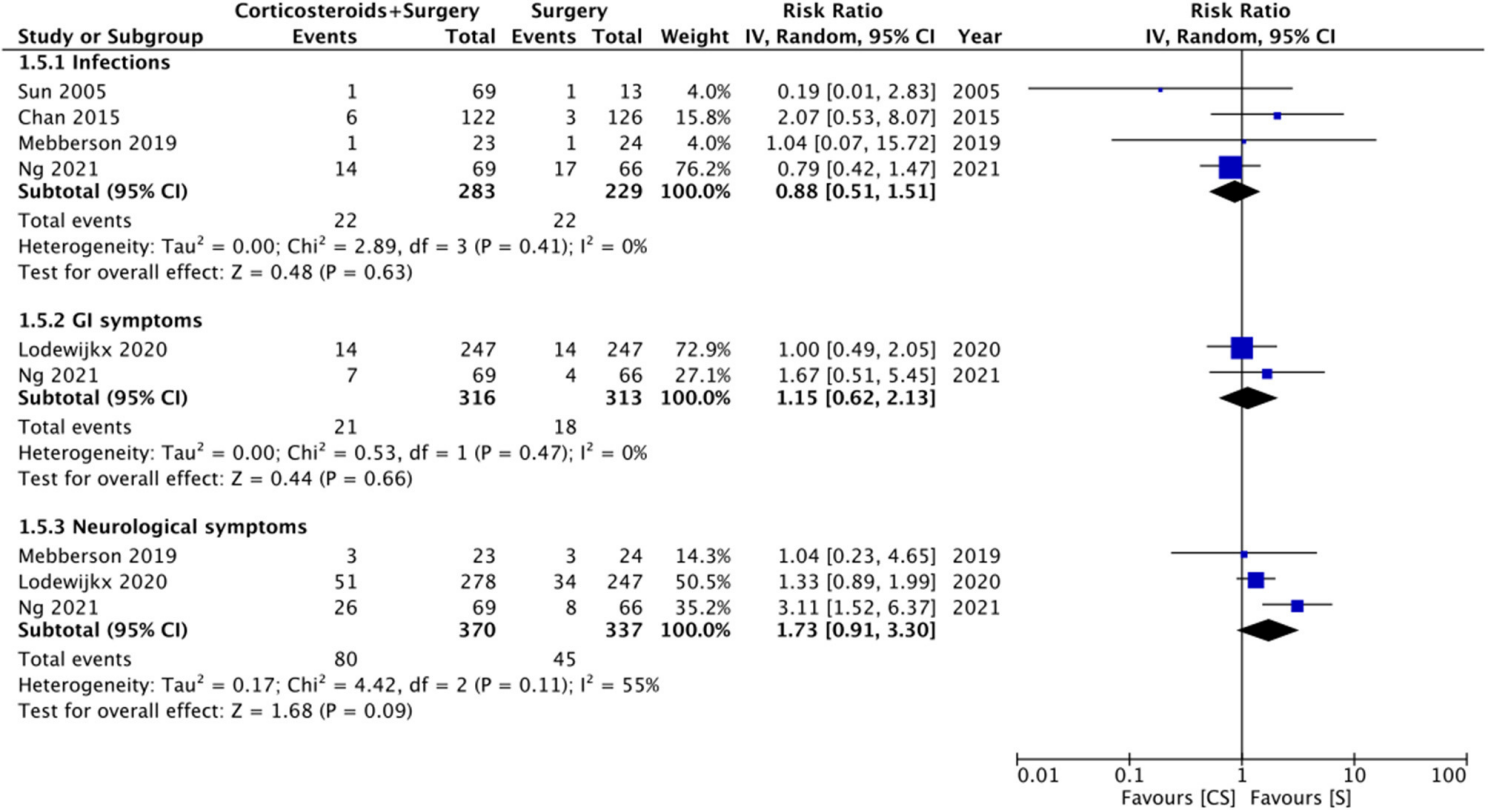
Meta-analysis of complications between corticosteroid and surgery (CS) and surgery (S) groups.

Limited data were available for different complications (Figure 6). Meta-analysis indicated no statistically significant differences in the risk of infections with the use of adjuvant corticosteroids (RR: 0.88 95% CI: 0.51, 1.51 *I*^2^ = 0% *p* = 0.63). Similarly, we noted no difference in the risk of gastrointestinal (RR: 1.15 95% CI: 0.62, 2.13 *I*^2^ = 0% *p* = 0.66) or neurological (RR: 1.73 95% CI: 0.91, 3.30 *I*^2^ = 55% *p* = 0.09) symptoms between the two groups.

### Risk of Bias

Quality analysis of included studies is presented in Table 2. Three RCTs were of high quality with a low risk of bias across all domains. All non-RCTs had a high risk of bias for confounding variables and blinding of outcome assessment.

**Table 2:** Authors judgement of risk of bias in included studies.

**RCTs**						
**Study**	**Randomization process**	**Deviation from intended intervention**	**Missing outcome data**	**Measurement of outcomes**	**Selection of reported result**	**Overall risk of bias**
Chan et al. (22)	Low risk	Low risk	High risk	High risk	Low risk	High risk
Mebberson et al. (15)	Low risk	Low risk	Low risk	Low risk	Low risk	Low risk
Hutchinson et al. (19)	Low risk	Low risk	Low risk	Low risk	Low risk	Low risk
Ng et al. (13)	Low risk	Low risk	Low risk	Low risk	Low risk	Low risk
**Non-RCTs**						
**Study**	**Selection of participants**	**Confounding variables**	**Intervention measurements**	**Blinding of outcome assessment**	**Incomplete outcome data**	**Selective outcome reporting**
Sun et al. (25)	Low risk	High risk	Low risk	High risk	Low risk	Low risk
Dran et al. (23)	Low risk	High risk	Low risk	High risk	Low risk	Low risk
Delgado et al. (26)	Low risk	High risk	Low risk	High risk	Low risk	Low risk
Qian et al. (21)	Low risk	High risk	Low risk	High risk	Low risk	Low risk
Fountas et al. (20)	Low risk	High risk	Low risk	High risk	Low risk	Low risk
Cofano et al. (24)	Low risk	High risk	Low risk	High risk	Low risk	Low risk
Lodewijkx et al. (14)	Low risk	High risk	Low risk	High risk	Low risk	Low risk

*RCT, Randomized control trial*.

## Discussion

Surgical intervention either by burr-hole, twist-drill, or craniostomy has been the recommended treatment for CSDH when there are symptoms suggestive of brain compression (5). While there is consensus on the fact that evacuation of hematoma is essential, there is still a debate on what constitutes the best surgical procedure and on the most optimal perioperative and postoperative management protocol (6). Several researchers have focused on delineating non-modifiable factors, such as hyperdense hematoma, bilateral hematomas, separated hematoma, severe brain atrophy, postoperative pneumocephalus, and modifiable factors, such as subdural irrigation and drain placement, that are all associated with recurrence after surgical management of CSDH (27–31). Therapeutic agents like corticosteroids have also been used in some patients but without clear evidence of their efficacy. However, with the publication of a few recent RCTs and by means of our meta-analysis, we hope to provide clarification on this important subject.

Our analysis of the data from 2,922 patients showed that the use of adjuvant corticosteroids results in a statistically significant 41% reduction in the recurrence rate after surgical management of CSDH. Overall, the recurrence rate was 6.35% in the corticosteroid plus surgery group and 13.17% in the surgery-only group. However, our review failed to demonstrate any beneficial effects of adjuvant corticosteroids on neurological outcomes and mortality rates. The effect estimates remained non-significant based on the variable definitions of good neurological outcomes as well as based on the study type. The results of our review concur with the outcomes reported by the previous meta-analysis but with a significant increase in statistical power. Holl et al. (9) in their review comparing data from 777 patients in five studies reported a statistically significant reduced risk of reintervention with adjuvant corticosteroids (RR: 0.44 95% CI: 0.27, 0.72 *I*^2^ = 0% *p* = 0.001). Importantly, of five studies, included in their review, only one was an RCT. Furthermore, like our review, they noted no statistically significant difference in good neurological outcomes and mortality with the use of adjuvant corticosteroids.

It should be noted that the selection bias is an important and inherent drawback of cohort studies which can significantly skew the study results. Non-randomized allocation of treatment based on neurological conditions results in biased treatment effect estimates rendering the comparison unscientific. However, while RCTs provide high-quality evidence, cohort studies provide real-world data which can increase our understanding of the treatment effect. Therefore, the current review included both RCTs and non-RCTs to provide comprehensive results on the efficacy of adjuvant steroids. Our meta-analysis increases the credibility of current evidence by adding five more studies to the previous review and includes three high-quality RCTs. The advantages of adjuvant corticosteroids in reducing recurrence are reiterated by the fact that the results were statistically significant on subgroup analysis of both RCTs and non-RCTs. However, it is also important to note that recurrence rates can be influenced by many other confounding factors like modified Nakaguchi-Classification, volumetric analysis, subdural irrigation, drain placement, etc. (27–31). While the influence of such confounders may have been minimal in RCTs, these variables could have affected outcomes of non-RCTs.

While the mechanism by which corticosteroids improve recurrence rates in CSDH is not very clear, it is postulated that the anti-inflammatory and anti-angiogenic properties of corticosteroids inhibit the formation of granulation tissue which creates the capsule around the hematoma. Since this capsule has neoangiogenic potential, with several budding permeable capillaries, its inhibition could reduce re-bleeding (32–34). Indeed, the beneficial effects of corticosteroids have been utilized for the medical management of CSDH. Corticosteroids have been used as monotherapy without any surgical intervention for lower grades of CSDH (35). However, primary medical treatment can be questionable for symptomatic CSDH. In one of the excluded studies, Miah et al. (36) have compared initial dexamethasone therapy vs. primary surgery in a cohort of 120 symptomatic CSDH patients. The authors noted lower recurrence rates with initial dexamethasone therapy as compared to primary surgery but 83% of patients in the initial dexamethasone group required surgical intervention later. The corticosteroid group also had higher complication rates and prolonged duration of hospital stay. Holl et al. (9) in their review have also demonstrated lower recurrence rates with corticosteroids with surgery as compared to corticosteroid therapy alone. Due to this variability of the results, there is still a reluctance amongst neurosurgeons to use conservative methods for managing CSDH (37). In one of the included trials, Hutchinson et al. (19) randomized patients to dexamethasone or placebo, and the final treatment decision (surgical or conservative monitoring) was left to the clinicians in consultation with the patients. However, 94% of their patients underwent surgical intervention. Since only a small proportion of their patients did not undergo surgery, we decided to include this trial in our meta-analysis. Notably, on sensitivity analysis, the exclusion of this study did not change the significance of any of the outcomes.

The use of corticosteroids is not without associated complications. Long-term administration can lead to glucose dysregulation, infections, gastrointestinal (GI) ulceration and bleeding, and neurological symptoms like delirium (38). Such adverse events largely depend upon the protocol of administration of corticosteroids. In our review, the dosage and duration of corticosteroid therapy varied across the included studies, but most administered the drug for ≥2 weeks. Since studies report the median time to recurrence of CSDH to be 12–15 days, a 2 week therapy seems to be feasible to reduce recurrence (39). With the currently reported regimens, our analysis found no statistically significant difference in infections, GI, and neurological symptoms with the use of corticosteroids. These results should be interpreted with caution as the included data was very limited. Hutchinson et al. (19) in their large RCT have reported significantly higher serious adverse events like hyperglycemia, new-onset diabetes, new-onset psychosis, and infections with dexamethasone as compared to placebo. However, since they reported data only graphically, their trial could not be included in the meta-analysis. It may be possible that shorter regimens of corticosteroids may influence the incidence of these adverse events.

Our review has some limitations. Despite providing a significant update from the previous review, we could include just three new RCTs in our analysis. Two of these RCTs were of limited sample size and one of them included just 47 patients. One of the included RCT had a high risk of bias (22). Furthermore, a large part of our results was derived from non-RCTs and as mentioned earlier, these types of studies have inherent selection bias. Additionally, there was significant methodological heterogeneity amongst the included studies regarding the type of patients included, the type, dosage, and duration of corticosteroid therapy, use of drains, and definition of outcomes. These differences could have impacted the study results. Furthermore, the timing of corticosteroid therapy and the period between diagnosis and surgical intervention were not clear in most studies. Lastly, the follow-up duration was quite variable across studies which could have influenced outcomes.

To conclude, the results of our updated systematic review and meta-analysis suggest that the use of adjuvant corticosteroids with surgery might significantly reduce the recurrence rates in patients with CSDH. However, corticosteroid therapy has no impact on neurological outcomes or mortality rates. Data on complications is scarce to derive firm conclusions. Future studies should assess the impact of different corticosteroid regimens on patient outcomes. The upcoming studies should use standardized reporting of neurological outcomes with uniform follow-up duration. Based on the current evidence, we believe that corticosteroids may be used as an adjuvant therapy to surgery in patients with CSDH with an aim to reduce recurrence. However, clinicians should also be cautious of complications associated with long-term steroid therapy.

## Data Availability Statement

The original contributions presented in the study are included in the article/Supplementary Material, further inquiries can be directed to the corresponding author/s.

## Author Contributions

GT and JC conceived and designed the study and analyzed the data. BL and SF were involved in literature search and data collection. GT and BL wrote the paper. JC and SF reviewed and edited the manuscript. All authors read and approved the final manuscript.

## Funding

The current study was funded by Chenzhou City-level Science and Technology Plan Project (Grant number: ZDYF2020109).

## Conflict of Interest

The authors declare that the research was conducted in the absence of any commercial or financial relationships that could be construed as a potential conflict of interest.

## Publisher's Note

All claims expressed in this article are solely those of the authors and do not necessarily represent those of their affiliated organizations, or those of the publisher, the editors and the reviewers. Any product that may be evaluated in this article, or claim that may be made by its manufacturer, is not guaranteed or endorsed by the publisher.

## Supplementary Material

Supplementary Figure 1 Funnel plot for the meta-analysis of good neurological outcome.

Supplementary Figure 2 Funnel plot for the meta-analysis of recurrence rates.

Supplementary Figure 3 Funnel plot for the meta-analysis of mortality rates.

Supplementary Table 1 Search strategy.
