# Supplementary figures and images for: The Efficacy of Adjuvant Corticosteroids in Surgical Management of Chronic Subdural Hematoma: A Systematic Review and Meta-Analysis
Source: Front Neurol. 2022 Jan 13;12:744266. doi: 10.3389/fneur.2021.744266 (PMC8792049; doi:10.3389/fneur.2021.744266)

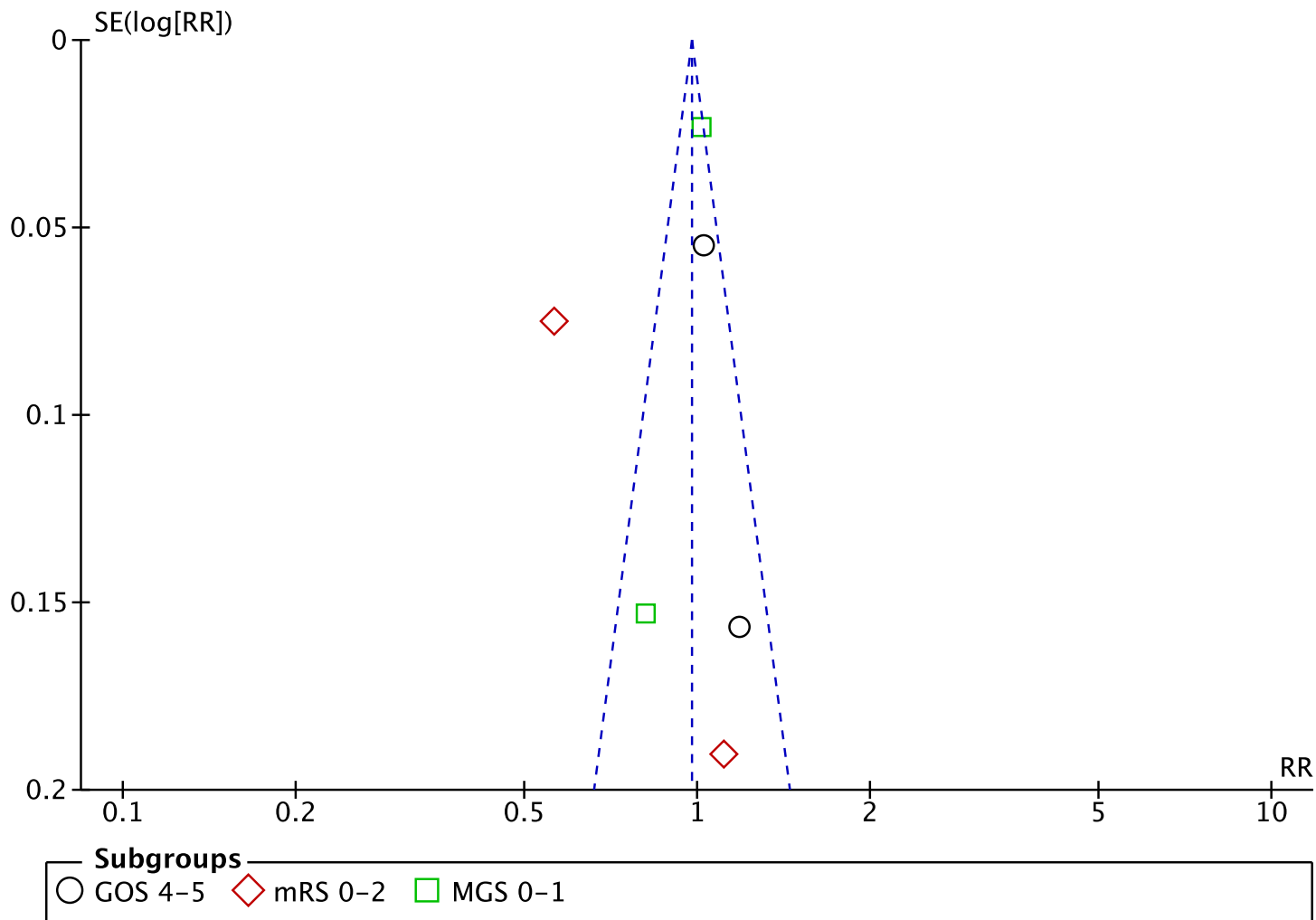

Supplement: Supplementary Figure 1 — Funnel plot for the meta-analysis of good neurological outcome. [file Data_Sheet_1.PDF]

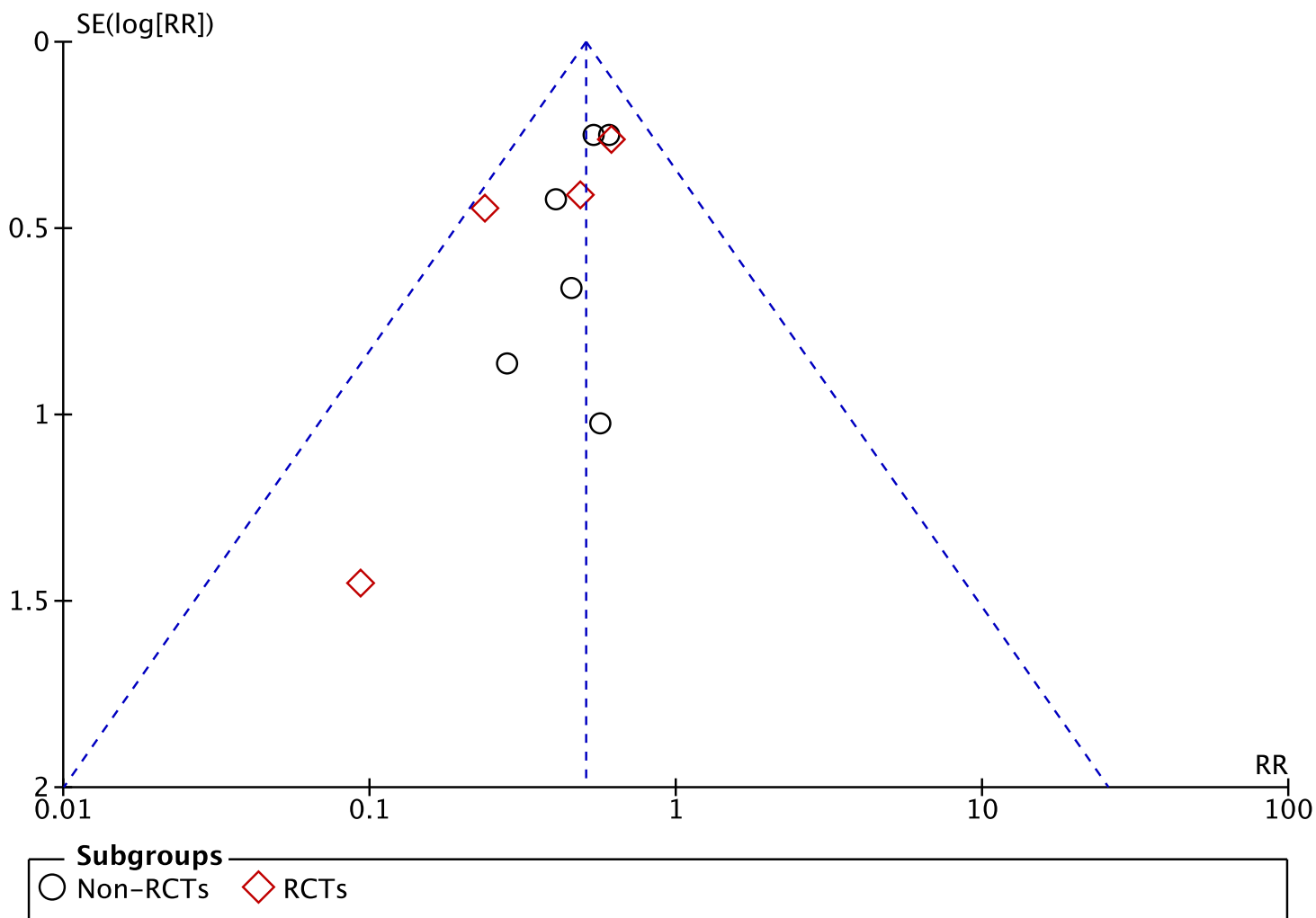

Supplement: Supplementary Figure 2 — Funnel plot for the meta-analysis of recurrence rates. [file Data_Sheet_2.PDF]

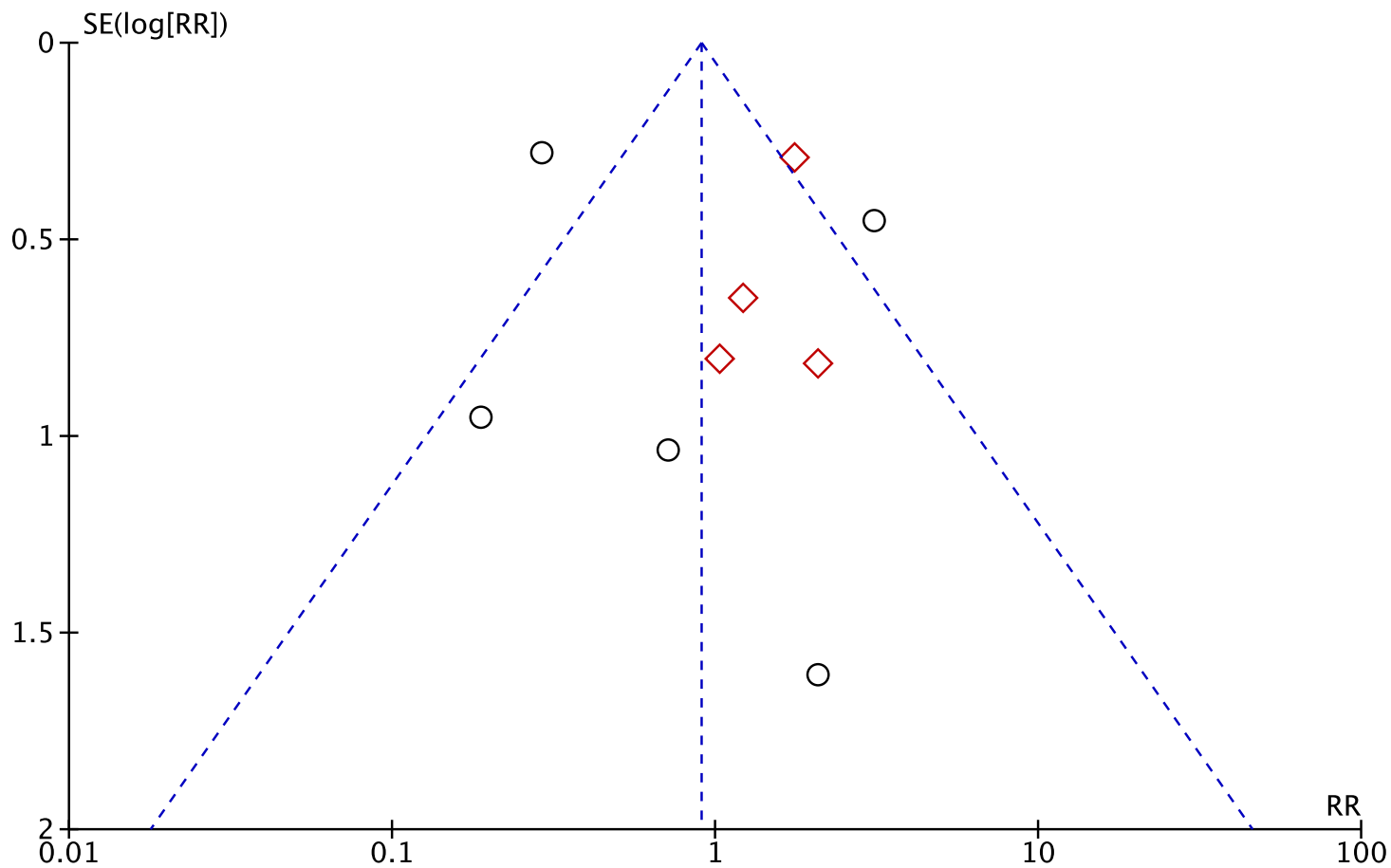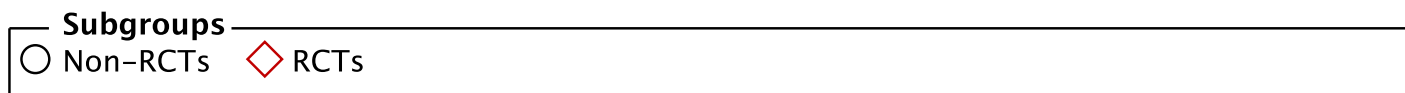

Supplement: Supplementary Figure 3 — Funnel plot for the meta-analysis of mortality rates. [file Data_Sheet_3.PDF]
