# Supplementary material for: The Efficacy of Adjuvant Corticosteroids in Surgical Management of Chronic Subdural Hematoma: A Systematic Review and Meta-Analysis
Source: Front Neurol. 2022 Jan 13;12:744266. doi: 10.3389/fneur.2021.744266 (PMC8792049; doi:10.3389/fneur.2021.744266)
Supplement: Supplementary Table 1 — Search strategy. [file Table_1.DOCX]

Supplementary Table 1: Search strategy

| **Search number** | **Query** | **Search Details** |
| --- | --- | --- |
| **1** | (((corticosteroids) OR (dexamethasone)) OR (prednisone)) AND (chronic subdural hematoma) | ("adrenal cortex hormones"[MeSH Terms] OR ("adrenal"[All Fields] AND "cortex"[All Fields] AND "hormones"[All Fields]) OR "adrenal cortex hormones"[All Fields] OR "corticosteroid"[All Fields] OR "corticosteroids"[All Fields] OR "corticosteroidal"[All Fields] OR "corticosteroide"[All Fields] OR "corticosteroides"[All Fields] OR ("dexamethason"[All Fields] OR "dexamethasone"[MeSH Terms] OR "dexamethasone"[All Fields] OR "dexamethasone s"[All Fields] OR "dexamethasones"[All Fields]) OR ("prednison"[All Fields] OR "prednisone"[MeSH Terms] OR "prednisone"[All Fields])) AND ("chronic subdural haematoma"[All Fields] OR "hematoma, subdural, chronic"[MeSH Terms] OR ("hematoma"[All Fields] AND "subdural"[All Fields] AND "chronic"[All Fields]) OR "chronic subdural hematoma"[All Fields] OR ("chronic"[All Fields] AND "subdural"[All Fields] AND "hematoma"[All Fields])) |
| **2** | (((corticosteroids) OR (dexamethasone)) OR (prednisone)) AND (intracranial hemorrhage) | ("adrenal cortex hormones"[MeSH Terms] OR ("adrenal"[All Fields] AND "cortex"[All Fields] AND "hormones"[All Fields]) OR "adrenal cortex hormones"[All Fields] OR "corticosteroid"[All Fields] OR "corticosteroids"[All Fields] OR "corticosteroidal"[All Fields] OR "corticosteroide"[All Fields] OR "corticosteroides"[All Fields] OR ("dexamethason"[All Fields] OR "dexamethasone"[MeSH Terms] OR "dexamethasone"[All Fields] OR "dexamethasone s"[All Fields] OR "dexamethasones"[All Fields]) OR ("prednison"[All Fields] OR "prednisone"[MeSH Terms] OR "prednisone"[All Fields])) AND ("intracranial haemorrhage"[All Fields] OR "intracranial hemorrhages"[MeSH Terms] OR ("intracranial"[All Fields] AND "hemorrhages"[All Fields]) OR "intracranial hemorrhages"[All Fields] OR ("intracranial"[All Fields] AND "hemorrhage"[All Fields]) OR "intracranial hemorrhage"[All Fields]) |
| **3** | (((surgery) OR (craniostomy)) OR (burr-hole)) AND (chronic subdural hematoma) | ("surgery"[MeSH Subheading] OR "surgery"[All Fields] OR "surgical procedures, operative"[MeSH Terms] OR ("surgical"[All Fields] AND "procedures"[All Fields] AND "operative"[All Fields]) OR "operative surgical procedures"[All Fields] OR "general surgery"[MeSH Terms] OR ("general"[All Fields] AND "surgery"[All Fields]) OR "general surgery"[All Fields] OR "surgery s"[All Fields] OR "surgerys"[All Fields] OR "surgeries"[All Fields] OR ("craniostomies"[All Fields] OR "craniostomy"[All Fields]) OR ("trephining"[MeSH Terms] OR "trephining"[All Fields] OR ("burr"[All Fields] AND "hole"[All Fields]) OR "burr hole"[All Fields])) AND ("chronic subdural haematoma"[All Fields] OR "hematoma, subdural, chronic"[MeSH Terms] OR ("hematoma"[All Fields] AND "subdural"[All Fields] AND "chronic"[All Fields]) OR "chronic subdural hematoma"[All Fields] OR ("chronic"[All Fields] AND "subdural"[All Fields] AND "hematoma"[All Fields])) |
